# Identification of Nasal Bone Fractures on Conventional Radiography and Facial CT: Comparison of the Diagnostic Accuracy in Different Imaging Modalities and Analysis of Interobserver Reliability

**DOI:** 10.5812/iranjradiol.6353

**Published:** 2013-08-30

**Authors:** Hye Jin Baek, Dong Wook Kim, Ji Hwa Ryu, Yoo Jin Lee

**Affiliations:** 1 Department of Radiology, Haeundae Paik Hospital, Inje University College of Medicine, Busan, South Korea; 2 Department of Radiology, Busan Paik Hospital, Inje University College of Medicine, Busan, South Korea

**Keywords:** Nasal Bone, Fractures, Bone, Radiography

## Abstract

**Background:**

There has been no study to compare the diagnostic accuracy of an experienced radiologist with a trainee in nasal bone fracture.

**Objectives:**

To compare the diagnostic accuracy between conventional radiography and computed tomography (CT) for the identification of nasal bone fractures and to evaluate the interobserver reliability between a staff radiologist and a trainee.

**Patients and Methods:**

A total of 108 patients who underwent conventional radiography and CT after acute nasal trauma were included in this retrospective study. Two readers, a staff radiologist and a second-year resident, independently assessed the results of the imaging studies.

**Results:**

Of the 108 patients, the presence of a nasal bone fracture was confirmed in 88 (81.5%) patients. The number of non-depressed fractures was higher than the number of depressed fractures. In nine (10.2%) patients, nasal bone fractures were only identified on conventional radiography, including three depressed and six non-depressed fractures. CT was more accurate as compared to conventional radiography for the identification of nasal bone fractures as determined by both readers (P <0.05), all diagnostic indices of an experienced radiologist were similar to or higher than those of a trainee, and κ statistics showed moderate agreement between the two diagnostic tools for both readers. There was no statistical difference in the assessment of interobserver reliability for both imaging modalities in the identification of nasal bone fractures.

**Conclusion:**

For the identification of nasal bone fractures, CT was significantly superior to conventional radiography. Although a staff radiologist showed better values in the identification of nasal bone fracture and differentiation between depressed and non-depressed fractures than a trainee, there was no statistically significant difference in the interpretation of conventional radiography and CT between a radiologist and a trainee.

## 1. Background

The nasal bone being a small, thin bone is the most common site of facial bone fracture. Nasal bone fractures have increased in prevalence and severity concurrently with the increase in traumas and traffic accidents (1-7). Accurate diagnosis and appropriate management of nasal bone fractures are important since fractures may cause conspicuous nasal deformities and functional problems of the nasal cavity (1). Many classifications of nasal bone fractures have been introduced; however, a standard classification has not yet been established (2, 3, 7, 8).

A nasal bone fracture is usually diagnosed by clinical examination and conventional radiography as the standard procedure. However, some investigators have reported the limitations of conventional radiography for the evaluation of nasal bone fractures (2, 4-6). Computed tomography (CT) has been shown to be a more accurate diagnostic tool than conventional radiography for evaluating nasal bone fractures and combined facial injuries (3, 6-11). The use of reformatted CT images, which reconstruct the original CT images to view different angles than the ones imaged, is important for the assessment of nasal bone and other facial bone fractures (3). However, it is currently unknown if CT can accurately diagnose nasal bone fractures better than conventional radiography when they are directly compared, and whether these results would differ between a trained radiographer and a lesser experienced trainee. To date, no objective study has shown a direct comparison of the diagnostic accuracy or interobserver reliability of conventional radiography and CT for the evaluation of nasal bone fractures.

## 2. Objectives

Therefore, one aim of our study was to directly compare the usefulness of conventional radiography and CT for the identification of nasal bone fractures. Another aim was to evaluate whether there are differences in the assessment between two individual readers (a staff radiologist and a trainee) in terms of their statistical interpretations of each of the conventional radiography and CT findings from nasal bone fractures.

## 3. Patients and Methods

### 3.1. Study Population

We retrospectively reviewed all facial CT examinations in the emergency department by using the database of our institution between January 2008 and June 2008: 378 patients (207 male and 171 female patients; age range, 5-88 years; mean age, 52.7 years) were selected. We then selected 145 of these 378 patients with the following inclusion criteria: patients with acute nasal trauma who had undergone both facial CT and conventional radiography (coronal and both lateral views) of the nasal bone on the same day by using electric medical chart and picture archiving and communication system (PACS). Of these 145 patients, 37 were excluded because of inadequate medical records (n=4), reduction of fractures before the studies (n=3), conventional radiography without lateral and coronal views of both nasal bones (n=13), facial CT images without sagittal and coronal reformatted images (n=7), or poor image quality, including motion artifacts or beam-hardening artifacts (n=10). The final 108 patients who were included in this study comprised 89 male and 19 female patients (age range, 10-72 years; mean, 35.4 years). The Institutional Review Board at our institution approved this study and determined that patient approval and informed consent were not required for reviewing images and records.

### 3.2. Radiological Imaging

The radiological examination consisted of coronal and lateral views of both nasal bones and a CT scan with axial images and coronal and sagittal reformatted images. These imaging sets were obtained on the same day in all patients. The nasal coronal view was obtained by placing the film cassette under the patient’s chin such that the plane of the film was at 37° angles to the orbitomeatal line. The nasal lateral view was obtained with the infraorbitomeatal line parallel to the transverse axis of the film and the intrapupillary line perpendicular to the plate. This orientation provided a true lateral projection that was neither tilted nor rotated. Conventional radiography was obtained at 50 kVp and 10 mA with a digital radiography system (DRS-800; Listem, Wonju, Gangwon-do, South Korea). Facial CT scanning was performed with settings of 120 kVp and 300 mA (window width, 2000 HU; window level, 350 HU), using a 16-channel multi-detector CT scanner (LightSpeed 16; GE Healthcare, Milwaukee, WI). The nasal bones of all the patients were scanned without using a contrast medium (slice thickness, 3 mm; reconstruction increment, 2 mm).

### 3.3. Image Analysis and Reference Standard

Short lucent lines that reached the anterior cortex of the nasal bone, with or without displacement, were regarded as fractures in both imaging modalities. Two readers, blinded to all patient information except for trauma, independently interpreted the radiographs and CT images of all patients. The readers interpreted the radiographs and CT images of the same patient on different days. The 2 readers were as follows: a staff radiologist with 8 years of experience in the diagnosis of head and neck imaging (reader 1) and a second-year resident (reader 2). Both readers classified the image findings into the following 3 groups: no fracture, depressed fracture, and non-depressed fracture. The image interpretation results were unified according to the agreement and disagreement between the two readers as follows: (1) the same results between the two readers (agreement) and (2) the final results after discussion between the two readers in the disagreement.

The consensus based on clinicomedical and intraoperative records between the two readers was used as the reference standard of nasal bone trauma. A pseudolesion, which mimicked a nasal bone fracture, was determined by consensus of the two readers, medical chart review, and operative notes.

### 3.4. Statistical Analysis

Statistical analyses were performed using SPSS 12.0 for Windows software (SPSS, Chicago, IL) and P<0.05 was considered statistically significant. The McNemar test and κ statistics were used to compare the results between the two imaging modalities and between the two readers. The results of κ statistics were interpreted as follows: κ values ranging from 0.21 to 0.40 indicated fair agreement; 0.41 to 0.60, moderate agreement; 0.61 to 0.80, good agreement; and 0.80 to 1.00, very good agreement. The diagnostic index (sensitivity, specificity, positive and negative predictive values, and accuracy) was calculated.

## 4. Results

**Figure 1.**
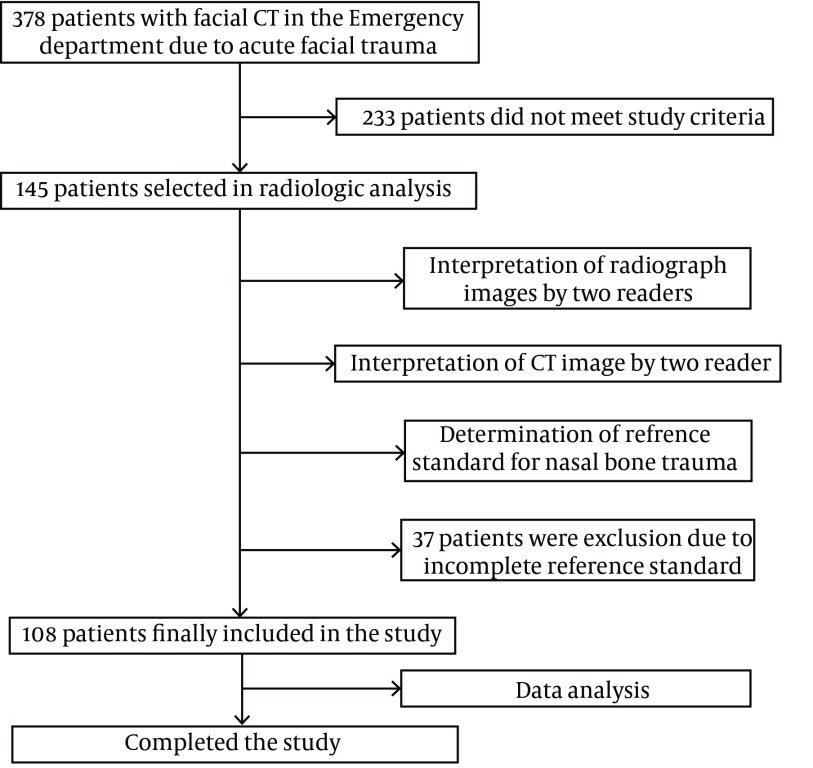
A diagram presenting enrollment of the studied patients and the study algorithm.

A diagram presenting enrollment of study patients and study algorithm is shown in Figure 1. 

Combined use of reformatted CT and conventional radiography is necessary to detect all types of nasal bone fractures.

Of the 108 patients, 88 (81.5%) were diagnosed with nasal bone fracture (72 men, 16 women; mean age, 35.7 years; age range, 20-70 years). A fracture line was found in at least one site of the nasal pyramid of 88 patients. The causes of nasal bone fractures were punches (n=28, 31.8%), traffic accidents (n=27, 30.7%), a slip or fall (n=17, 19.3%), sports injury (n=9, 10.2%), and work-related injuries (n=7, 8.0%). The cases included only nasal bone fracture (n=48), nasal bone fracture associated with another facial bone fracture (n = 29), and nasal bone fracture associated with skull fracture (n = 11). The main sites of associated facial bone fractures were the orbit (n=19), maxilla (n=4), zygoma (n=3), and mandible (n=3).

**Figure 2.**
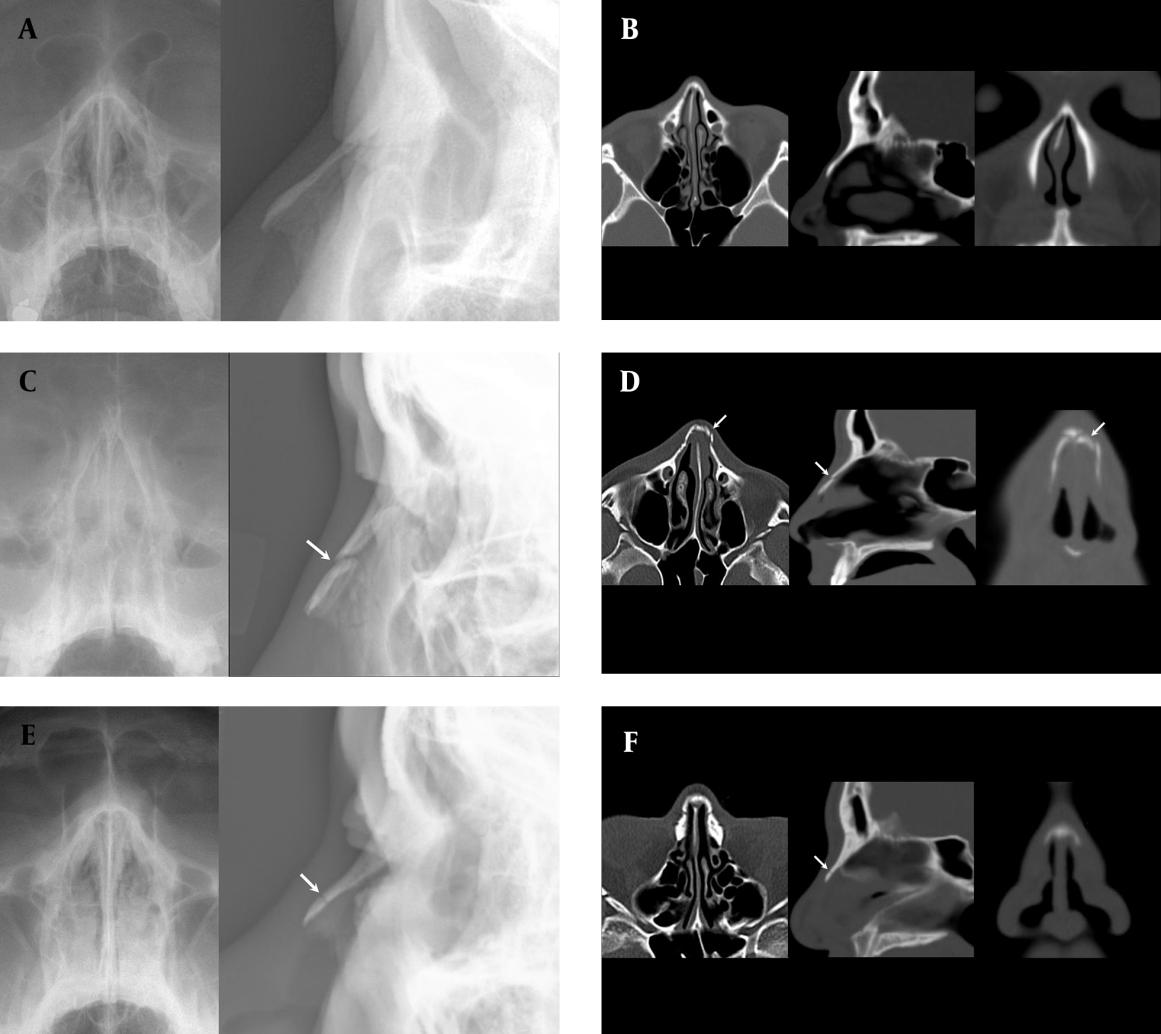
Three slices of the nasal bone are shown: A and B, a 28-year-old man with no fracture (A: coronal and lateral conventional radiography images, B: axial, sagittal, and coronal reformatted CT images). C and D, a 24-year-old man with depressed fracture (C: coronal and lateral conventional radiography images, D: axial, sagittal, and coronal reformatted CT images). E and F, a 42-year-old man with non-depressed fracture (E: coronal and lateral conventional radiography images, F: axial, sagittal, and coronal reformatted CT images) (arrows indicate nasal bone fracture).

The incidence of non-depressed fractures (59/88, 67.0%) was higher than that of depressed fractures (29/88, 33.0%) (Figure 2). The distal portion of the nasal bone was the most common fracture site (n=60, 68.2%). The 88 cases were categorized as follows: simple fracture without displacement (n=13, 14.8%); simple fracture with displacement (n=61, 69.3%), including unilateral fracture without septal fracture (n=7); unilateral fracture with septal fracture (n=8); bilateral fracture without septal fracture (n=29); bilateral fracture with septal fracture (n=17); and comminuted fracture (n=14, 15.9%). Of the 88 patients with a nasal bone fracture, 67 (76.1%) underwent surgery for nasal reduction. The remaining patients did not undergo surgical intervention because of the patient refusal or the presence of only a minimal fracture without a significant nasal deformity, which did not require operative correction. 

**Figure 3:**
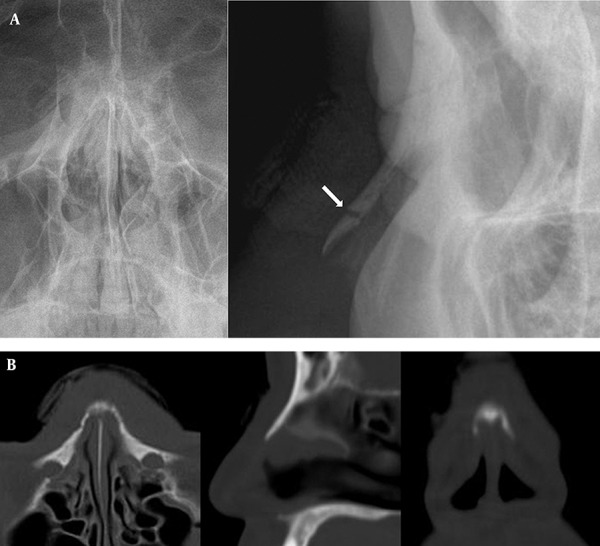
A 36-year-old man with painful nasal swelling and a simple non-depressed transverse nasal fracture. A, Coronal and lateral conventional radiography images show a discrete simple fracture in the mid-portion of the nasal bone. B, CT images show no discrete fracture on axial, sagittal, and coronal reformatted images (arrow indicates nasal bone fracture).

Out of 88 patients, 79 (89.8%) had nasal bone fractures which were detected on CT, and 58 patients (65.9%) showed fractures on both CT and conventional radiography. For the overall detection of nasal bone fractures, all values indicated the significant superiority of CT over conventional radiography (McNemar test, P<0.05) (Table 1). In nine (10.2%) patients, nasal bone fractures were identified on only conventional radiography, including three depressed and six non-depressed fractures (Figure 3). This result indicated that conventional radiography detected fractures that were missed by CT imaging. 

**Table 1.** Diagnostic Indices of Conventional Radiography and CT for Overall Nasal Bone Fracture

	Sensitivity, (%)	Specificity, (%)	PPV, (%)	NPV, (%)	Accuracy, (%)
Radiography*	67/88(76.1)	4/20(20)	67/83(80.7)	4/25(16)	71/108(65.7)
CT*	79/88(89.8)	18/20(90)	79/81(97.5)	18/27(66.7)	97/108(89.8)

Abbreviations: PPV, Positive Predictive Value; NPV, Negative Predictive Value *Diagnostic indices of radiography and CT were calculated on the basis with the consensus between two radiologists.

Image readings between two independent readers were not significantly different while there were moderate disagreements in interpretation using the two different imaging modalities. The reference standards were determined by consensus of two readers on the basis of the imaging features and medical chart review, including clinical examination and intraoperative findings. Diagnostic indices of reader 1 and reader 2 for overall nasal bone fracture on conventional radiography and CT are summarized in Table 2. For reader 1, there was no significant difference in all statistic values between conventional radiography and CT (P>0.05, McNemar test). For reader 2, there was no significant difference in specificity between conventional radiography and CT (P=0.16, McNemar test), whereas there was a significant difference in the other values between conventional radiography and CT (P<0.05, McNemar test). The specificity and positive predictive value of CT in both readers were both 100%, but most values in reader 1 were higher than those of reader 2 in both imaging tools. 

**Table 2.** Diagnostic Indices of Reader 1 and Reader 2 for Overall Nasal Bone Fracture on Conventional Radiography and CT

		Reference Standard		Total,(n)	Sensitivity,(%)	Specificity,(%)	PPV,(%)	NPV,(%)	Accuracy,(%)
		Fx (n)	NF (n)						
Reader 1* Radiography	**Fx**	76	1	77	76/88 (86.4)	19/20 (95)	76/77 (98.7)	19/31 (61.3)	95/108 (88)
	**NF**	12	19	31					
Reader 2** Radiography	**Fx**	67	1	68	67/88 (76.1)	19/20 (95)	67/68 (98.5)	19/40 (47.5)	86/108 (79.6)
	**NF**	21	19	40					
Reader 1* CT	**Fx**	79	0	79	79/88 (89.8)	20/20 (100)	79/79 (100)	20/29 (68.8)	99/108 (91.7)
	**NF**	9	20	29					
Reader2** CT	**Fx**	80	0	80	80/88 (90.9)	20/20 (100)	80/80 (100)	20/28 (71.4)	100/108 (92.6)
	**NF**	8	20	28					
**Total**		88	20	108					

Abbreviations: Fx, Fracture; NF, No Fracture; PPV, Positive Predictive Value; NPV, Negative Predictive Value Reader 1*: an experienced radiologist, Reader 2**: a trainee

When the fractures were subdivided into two types (depressed and non-depressed), the comparison of these subtypes by both readers revealed that most values obtained with CT were superior to those obtained with radiography (Table 3). The comparison of the results for the use of both imaging modalities between reader 1 and reader 2 according to the fracture type showed that all values of reader 1 were superior to those of reader 2. For composite data about depressed fracture, reader 1 shows no significant difference in all values between conventional radiography and CT (P>0.05, McNemar test), but reader 2 shows significant difference (P<0.05, McNemar test). For composite data about non-depressed fracture, both readers show no significant difference in all values between conventional radiography and CT (P>0.05, McNemar test). 

**Table 3.** Diagnostic Indices of Reader 1 and Reader 2 for Two Types of Nasal Bone Fracture on Conventional Radiography and CT

		Reference Standard			Total (n)	Sensitivity (%)	Specificity (%)	PPV(%)	NPV(%)	Accuracy (%)
		DPF (n)	NDPF (n)	NF (n)						
Reader 1* Radiography	**DPF**	15	4	0	19	61/84(72.6)	19/24(79.2)	61/66(92.4)	19/42(45.2)	80/108(74.1)
	**NDPF**	11	46	1	58					
	**NF**	3	9	19	31					
Reader 2** Radiography	**DPF**	19	9	0	28	52/79(65.8)	19/29(65.5)	52/62(83.9)	19/46(41.3)	71/108(65.7)
	**NDPF**	6	33	1	40					
	**NF**	4	17	19	40					
Reader 1* CT	**DPF**	26	2	0	28	77/86(89.5)	20/22(90.9)	77/79(97.5)	20/29(69.0)	97/108(89.8)
	**NDPF**	0	51	0	51					
	**NF**	3	6	20	29					
Reader 2** CT	**DPF**	24	20	0	44	58/68(85.3)	30/50(60)	58/78(74.4)	30/40(75)	88/108(81.5)
	**NDPF**	2	34	0	36					
	**NF**	3	5	20	28					
**Total**		29	59	20	108					

Abbreviations: DPF, Depressed Fracture; NDPF, Non-Depressed Fracture; NF, No Fracture; PPV, Positive Predictive Value; NPV, Negative Predictive Value Reader 1*: an experienced radiologist, Reader 2**: a trainee

The use of κ statistics showed moderate agreement between the two diagnostic tools for both readers (Table 4). In particular, CT shows higher value than conventional radiography. Interobserver reliability for detecting subtypes of nasal bone fracture between the two diagnostic tools for both readers is summarized in Table 5. The results of the two readers were not significantly different, since κ statistics showed good agreement (κ value, 0.629; P<0.001) for the use of conventional radiography findings and a moderate agreement (κ value, 0.570; P<0.001) for the use of CT findings. These results indicate that diagnosis based on CT or conventional radiography was similar regardless of the reader’s experience. 

**Table 4.** Interobserver Reliability for the Identification of Nasal Bone Fracture on Conventional Radiography and CT

	Reader 1*		Reader 2**		κ value(95% CI)	P value
	Fx(n)	NF (n)	Fx(n)	NF(n)		
**Radiography**	77	31	68	40	0.646 (0.495-0.797)	<0.001
**CT**	79	29	80	28	0.833 (0.713-0.953)	<0.001

Abbreviations: Fx, Fracture; NF, No Fracture; CI, Confidence Interval Reader 1*: an experienced radiologist, Reader 2**: a trainee

**Table 5.** Interobserver Reliability for Detecting Subtypes of Nasal Bone Fracture on Conventional Radiography and CT

	Reader 1*			Reader 2**			κ value(95% CI)	P value
	DPF (n)	NDPF (n)	NF (n)	DPF (n)	NDPF (n)	NF (n)		
**Radiography**	19	58	31	28	40	40	0.629 (0.507-0.751)	<0.001
**CT**	28	51	29	44	36	28	0.570 (0.443-0.697)	<0.001

Abbreviations:DPF, Depressed Fracture; NDPF, Non-Depressed Fracture; NF, No Fracture; CI, Confidence Interval Reader 1*: an experienced radiologist, Reader 2**: a trainee

## 5. Discussion

Nasal bone fracture is the most common type of facial bone fracture, and approximately 50% of facial fractures are isolated fractures of the nasal pyramid (8-12). Hwang et al.(3) reviewed 503 cases by analyzing nasal bone fractures by conventional radiography in both lateral and Waters views, as well as by CT. Only 82% of nasal bone fractures were identified by conventional radiography vs. 100% by CT. In this study, diagnostic accuracy of facial CT was superior to that of conventional radiography in the detection of nasal bone fracture. In particular, facial CT showed a high positive predictive value unlike conventional radiography. Plain films were deemed unreliable for the diagnosis of nasal bone fractures. In our study, conventional radiography showed limited diagnostic accuracy of nasal bone fractures because of the presence of several pseudolesions, such as prior nasal bone fracture, angulation deformity, anatomical variation, fracture of ossified cartilage, midline nasal suture or nasomaxillary suture, a thin nasal wall, and Mach band artifact (eyelid or vascular marking). However, for nine (10.2%) patients, including 3 patients with simple depressed fracture and 6 with non-depressed fracture, the fracture was identified only on a conventional radiograph. Although conventional radiography should not be used as the sole diagnostic tool, it is a useful complementary imaging tool for the detection of transverse fractures of the nasal bone. Nevertheless, facial CT using 1.5 mm or less slice thickness may detect non-depressed nasal fracture, and further studies are required.

Several studies have suggested that CT is very useful for the diagnosis of nasal bone fracture (6-9, 11). In cases of very severe injuries, a higher detection rate and better clinical correlation were achieved with CT (8). In our study, CT was significantly superior to conventional radiography for the detection of nasal bone fractures. Furthermore, there was no significant difference between the abilities of the trainee and the radiologist identifying nasal bone fractures revealed by both imaging modalities. Therefore, our data suggest that the identification of nasal bone fractures by conventional radiographs or CT images does not necessarily require an extensively trained radiologist. In the diagnosis of depressed or non-depressed fracture, however, a staff radiologist showed better values than a trainee in both imaging tools.

Facial CT is commonly used in an acute trauma setting to obtain information since it is the most preferred modality for complete evaluation of injuries of the facial skeleton and facial soft tissues (3, 6-9, 11). However, facial CT may provide inadequate information by imaging prior fractures, normal sutures, or other non-traumatic abnormalities, which could be misleading. In this study, nine cases of simple transverse fractures in the presence or absence of mild depressed were undetected on facial CT, while the fractures were clearly identified by conventional radiography. We conclude that conventional radiographs, including both lateral views of the nasal bone, are superior to sagittal reformatted CT images with 3 mm slice thickness. To the best of our knowledge, a study of CT imaging with a slice thickness of 1.5 mm or less, for nasal bone fractures, has not been performed. We believe that a further study of sagittal reformatted CT images of slice thickness of 1.5 mm or less is required.

Although the findings of this study might be useful in determining the best evaluation method for nasal bone fractures, the study has several limitations. First, most of the cases were multiple or complicated fractures, limiting the data that could be obtained from simple fractures. Second, the data collected were not compared to previously defined classifications according to injury mechanism, injury location, or configuration of a fracture. In addition, the slice thickness of 3 mm for reformatted CT images was used because it was the standard in the emergency department of our institution. However, higher precision images, which give more information, might be acquired using a slice thickness of 1 mm or less; therefore, further studies using thinner slices are necessary to best compare the different imaging methods. Finally, the consensus between two readers in the disagreement of image analysis was not objective.

In summary, CT is superior to conventional radiography for the detection of nasal bone fractures, assessment of the type of nasal bone fracture, for combined injuries, and for decision-making in therapeutic planning. However, although conventional radiography is not the first choice as a diagnostic tool, it may be useful for the detection of transverse and non-depressed nasal bone fractures. Moreover, the identification of nasal bone fractures by a radiologist and a trainee were not significantly different using conventional radiographs and CT for diagnosis, whereas a staff radiologist showed better values in the identification of nasal bone fracture and differentiation between depressed and non-depressed fractures than a trainee.

## References

[1] Tremolet de Villers Y (1975). Nasal fractures.. J Trauma..

[2] Thiede O, Kromer JH, Rudack C, Stoll W, Osada N, Schmal F (2005). Comparison of ultrasonography and conventional radiography in the diagnosis of nasal fractures.. Arch Otolaryngol Head Neck Surg..

[3] Hwang K, You SH, Kim SG, Lee SI (2006). Analysis of nasal bone fractures; a six-year study of 503 patients.. J Craniofac Surg..

[4] Nigam A, Goni A, Benjamin A, Dasgupta AR (1993). The value of radiographs in the management of the fractured nose.. Arch Emerg Med..

[5] Clayton MI, Lesser TH (1986). The role of radiography in the management of nasal fractures.. J Laryngol Otol..

[6] Ziccardi VB, Braidy H (2009). Management of nasal fractures.. Oral Maxillofac Surg Clin North Am..

[7] Yabe T, Ozawa T, Sakamoto M, Ishii M (2004). Pre- and postoperative x-ray and computed tomography evaluation in acute nasal fracture.. Ann Plast Surg..

[8] Som PM, Curtin HD (2011). Head and Neck Imaging: Expert Consult- Online and Print..

[9] Manson PN, Markowitz B, Mirvis S, Dunham M, Yaremchuk M (1990). Toward CT-based facial fracture treatment.. Plast Reconstr Surg..

[10] de Lacey GJ, Wignall BK, Hussain S, Reidy JR (1977). The radiology of nasal injuries: problems of interpretation and clinical relevance.. Br J Radiol..

[11] Hirota Y, Shimizu Y, Iinuma T (1988). [Image diagnosis of nasal bone fracture].. Nihon Jibiinkoka Gakkai Kaiho..

[12] Logan M, O'Driscoll K, Masterson J (1994). The utility of nasal bone radiographs in nasal trauma.. Clinic Radiol..

